# Evaluation of *Staphylococcus aureus* Eradication Therapy in Vascular Surgery

**DOI:** 10.1371/journal.pone.0161058

**Published:** 2016-08-16

**Authors:** J. C. M. Langenberg, A. R. Thomas, J. M. W. Donker, M. M. L. van Rijen, J. A. J. W. Kluytmans, L. van der Laan

**Affiliations:** 1 Department of Surgery, Amphia Hospital, Breda, The Netherlands; 2 Laboratory of Microbiology and Infection Control, Amphia Hospital, Breda, The Netherlands; 3 Julius Center for Health Sciences and Primary Care, UMCU, Utrecht, The Netherlands; Chang Gung Memorial Hospital, TAIWAN

## Abstract

**Introduction:**

Surgical site infections (SSI) are a serious complication in vascular surgery which may lead to severe morbidity and mortality. *Staphylococcus aureus* nasal carriage is associated with increased risk for development of SSIs in central vascular surgery. The risk for SSI can be reduced by perioperative eradication of *S*. *aureus* carriage in cardiothoracic and orthopedic surgery. This study analyzes the relation between *S*. *aureus* eradication therapy and SSI in a vascular surgery population.

**Methods:**

A prospective cohort study was performed, including all patients undergoing vascular surgery between February 2013 and April 2015. Patients were screened for *S*. *aureus* nasal carriage and, when tested positive, were subsequently treated with eradication therapy. The presence of SSI was recorded based on criteria of the CDC. The control group consisted of a cohort of vascular surgery patients in 2010, who were screened, but received no treatment.

**Results:**

A total of 444 patients were screened. 104 nasal swabs were positive for *S*. *aureus*, these patients were included in the intervention group. 204 patients were screened in the 2010 cohort. 51 tested positive and were included in the control group. The incidence of *S*. *aureus* infection was 5 out of 51 (9.8%) in the control group versus 3 out of 104 in the eradication group (2.2%; 95% confidence interval 0.02–1.39; P = 0.13). A subgroup analysis showed that the incidence of *S*. *aureus* infection was 3 out of 23 (13.0%) in the control group in central reconstructive surgery versus 0 out of 44 in the intervention group (P = 0.074). The reduction of infection pressure by *S*. *aureus* was stronger than the reduction of infection pressure by other pathogens (exact maximum likelihood estimation; OR = 0.0724; 95% CI: 0.001–0.98; p = 0.0475).

**Conclusion:**

*S*. *aureus* eradication therapy reduces the infection pressure of *S*. *aureus*, resulting in a reduction of SSIs caused by *S*. *aureus*.

## Introduction

*Staphylococcus aureus* nasal carriage is associated with an increased risk for developing a health care-related infection with this micro-organism. This effect has been described in several populations, e.g. cardiac surgery, orthopedic surgery and peritoneal dialysis [1–3]. Preoperative screening and subsequent treatment of nasal *S*. *aureus* carriers with mupirocin and chlorhexidine reduces the risk of developing hospital-acquired infections as well as the duration of hospital stay [4, 5]. A cost benefit analysis shows that this strategy is highly cost-effective [6].

In vascular surgery the relation between nasal carriage of *S*. *aureus* and the development of surgical site infections (SSI) has been found for patients undergoing central vascular reconstruction surgery. In other peripheral vascular procedures this relation has not been demonstrated [7]. Little is known about the result of treatment of carriage in the field of vascular surgery, even though it has been shown that surgical site infections are related to serious morbidity and mortality [8, 9]. This study evaluates the effect of nasal screening and subsequent eradication therapy in vascular surgical procedures. The aim of this study is to investigate whether nasal screening and eradication of *S*. *aureus* reduces the incidence of surgical site infections caused by this micro-organism.

## Patients and Methods

A clinical audit was performed on all patients undergoing vascular surgery between February 2013 and April 2015 in the Amphia Hospital, Breda, Netherlands, as part of a quality assurance study.

The surgical procedures included central reconstructions for abdominal aortic aneurysms and occlusive disease (endovascular [EVAR] and open), peripheral bypass procedures (autologous and polytetrafluoroethylene [PTFE]), endarterectomies of the femoral and carotid artery, embolectomies and artero-venous access procedures. All patients received 2 grams of cefazolin as standard preoperative antibiotics

Baseline characteristics including gender, age and risk factors such as diabetes mellitus, smoking, hypertension, end stage renal failure and congestive heart failure were obtained from all included patients. Type of surgery (central or peripheral) and the use of an implant were also assessed.

As described in detail previously [7], all patients were screened for *S*. *aureus* on the day that they were admitted to the vascular surgery department of our hospital. Screening was performed by using a dry, sterile swab, which was rotated four times in each nostril. The swab was placed in saline and subsequently centrifuged. Part of the sample was processed for polymerase chain reaction (PCR) on the presence of *S*. *aureus*, and part was inoculated onto a blood agar plate, to allow nasal and infecting strains to be compared in case a surgical site infection did occur.

The GeneXpert MRSA/SA Assay (Cepheid, Sunnyvale, CA) is a real-time PCR-based method, capable of both identifying *S*. *aureus* and differentiating whether a *S*. *aureus* is methicillin-susceptible (MSSA) or methicillin-resistant (MRSA) [10–13].

Patients who were tested positive for *S*. *aureus* were subsequently treated with eradication therapy.

Eradication therapy consisted of mupirocin ointment 2% (Bactroban, GlaxoSmithKline) in combination with chlorhexidine gluconate soap, 40mg per milliliter (Hibiscrub, Mölnycke). Bactroban ointment was applied twice a day, and Hibiscrub was used daily for a total body wash. The duration of the treatment was 5 days.

Patients were followed prospectively for the development of SSIs for at least 30 days. SSI was defined in agreement with the Centers for Disease Control’s criteria [14].

The main criteria are the presence of pain, heat, swelling and/or redness around the wound within 30 days after the initial procedure, as well as the presence of a positive culture, drainage of the wound or the presence of pus upon performing a diagnostic puncture. All infections were confirmed by a physician.

The control group consisted of a cohort of patients who underwent vascular surgery at the Amphia Hospital between January 1^st^ and December 31th 2010 and were screened for *S*. *aureus* nasal carriage and prospectively followed for the development of surgical site infections as described above, but who did not receive any eradication treatment.

Screening was performed as part of the infection control strategy of the Amphia hospital using non-invasive sampling and anonymized data analysis. Approval of the medical ethical committee and informed consent were not applicable in this evaluation of clinical practice.

Statistical analysis was performed using SPSS software v21.0 (SPSS Inc., Chicago, IL, USA)

And LogXact (Cytel, Cambridge, MA, USA). A binary logistic regression with a fisher’s exact test was used to determine significance. A P-value of < 0.05 was considered significant.

## Results

### Patient population

Between February 1st 2013 and April 31th 2015, 592 patients were admitted to the hospital who underwent vascular surgery. Of these patients, 148 patients were not screened due to emergency admissions or non-compliance of staff and patients. Of the 444 patients who were screened, 104 (23.4%) grew *S*. *aureus* and were subsequently treated. These patients are further indicated as the intervention group.

In the 2010 cohort, 326 patients were admitted to the hospital, of which 122 were not screened, 153 screened negative and 51 (25.0%) screened positive but were not treated. These 51 patients were considered as a historical control group.

### Patient characteristics

The percentage of ASA 2 patients was significantly lower in the intervention group. No differences were observed in the rest of baseline characteristics between the intervention and historical control group (Table 1).

**Table 1:** Baseline characteristics of the 155 study patient characteristics.

Characteristics	Screened positive, not treated (n = 51)	Screened positive, treated (n = 104)	P-value (Chi-square test)
Mean (SD) age	71.5 (10.6)	69.5 (8.6)	0.216
Sex, Male/Female (% male)	39/12 (77)	78/26 (73)	1.000
Peripheral procedure N/(%)	28 (55)	60 (58)	0.863
Implant used N/(%)	35 (69)	70 (67)	1.000
Risk factor N/(%)			
Mean (SD) Body Mass Index	24.8 (6.3)	26.0 (5.0)	0.222
Mean (SD) Length of surgery in minutes	171 (72)	151 (61)	0.084
ASA (%)			0.023
2	12 (24)	47 (46)	
3	36 (72)	51 (50)	
4	2 (4)	2 (4)	
Smoking	19 (37)	36 (34)	0.858
Diabetes mellitus	20 (39)	37 (35)	0.724
Hypertension	27 (53)	54 (52)	1.000
End Stage Renal failure	4 (8)	7 (7)	0.752
Congestive heart failure	24 (47)	46 (44)	0.864

### Infection rates

There were 6 (11.7%) surgical site infections in the non-treated group (n = 51) versus 13 (13.6%) in the treated group (n = 104) (OR 1.09, 95%CI 0.62–2.029 P = 0.97). In 8 of these surgical site infections, wound swabs tested positive for *S*. *aureus*. 5 (9.8%) occurred in the non-treated group versus 3 (2.2%) in the treated group (OR 0.37; 95%CI 0.02–1.39; P = 0.13) (Table 2). There were no cases of Methicillin resistant *S*. aureus (MRSA). The reduction of infection pressure by *S*. *aureus* is greater than the reduction of infection pressure by other pathogens (exact maximum likelihood estimation; OR = 0.0724; 95% CI: 0.001–0.98; p = 0.0475).

**Table 2:** Infection rates in intervention and control group.

		Positive, not treated (n = 51)	Positive, treated (n = 104)	Odds Ratio	95% CI; P value(chi-square test)
Total SSI (%)		6 (11.8)	13 (12.5)	1.09	0.62–2.029; P = 0.97
	Non *S*. *aureus* SSI (%)	1	10		
	Type				
	*Pseudomonas aeruginosa*	1			
	*Staphylococcus capitis*		1		
	*Escherichia coli*		1		
	*Proteus mirabilis*		1		
	*Morganella morganii*		1		
	*Enterobacter cloacae*		1		
	*Streptococcus anginosus*		1		
	*Unable to obtain culture (superficial infection)*		4		
	*S*. *aureus* SSI (%)	5 (9.8)	3 (2.8)	0.37	0.02–1.39; P = 0.13

### Stratified analysis of infection rate

In central reconstructive surgery (n = 67), no cases of *S*. *aureus* SSI appeared in the intervention group, opposed to 3 (13.0%) in the historical control group (p = 0.04, The exact one-sided p-value is used here, since the proper 95%-confidence interval corresponds with a one-sided 97.5%-confidence interval, because there is no lower limit [a value of 0 SSIs was found in the intervention group]. In this case, significance should be tested against an alpha-value of 0.025. Therefore, significance is not reached in this case.) In peripheral procedures, 3 (5.0%) *S*. *aureus* SSIs were encountered in the intervention group, compared to 2 (7.1%) *S*. *aureus* infections in the control group (P = 0.651) (Table 3).

**Table 3:** Stratified infection rates.

Surgery type			
		*S*. *aureus* infection rate	P-value (chi-square test)
Central reconstruction (n = 67)	Treated (n = 44)	0 (0.0%)	0.04
	Non treated (n = 23)	3 (13.0%)	
Peripheral procedure (n = 88)	Treated (n = 60)	3 (5.0%)	0.651
	Non treated (n = 28)	2 (7.1%)	
Implant (n = 105)	Treated (n = 70)	1 (1.4%)	0.041
	Non treated (n = 35)	4 (11.4%)	
No implant (n = 50)	Treated (n = 16)	1 (6.3%)	1.000
	Non treated (n = 34)	2 (5.9%)	

## Discussion

This clinical evaluation shows that surgical site infections are a relatively frequent complication in vascular surgery and that a large percentage (42%) is caused by *S*. *aureus*. Overall, there is no significant relation between eradication of *S*. *aureus* and the occurrence of surgical site infections. Analysis shows that the infection pressure by *S*. *aureus* is greatly reduced by eradication treatment in comparison to the overall infection pressure, while the general infection pressure does not seem to diminish. This results in a reduced incidence of *S*. *aureus* infections, while not lowering the overall SSI rate. This could be explained due to the competing nature of infection pathogens. When the growth and virulence of *S*. *aureus* are removed from the surgical site’s bacterial ‘pool’, other pathogens which do not react on our proposed eradication therapy are given the chance to flourish and cause a surgical site infection.

The infection pressure of *S*. *aureus* in vascular surgery can be significantly lowered by using mupirocin and chlorhexidine gluconate soap eradication therapy in *S*. *aureus* carriers. Recent research shows that a reduction in the amount of *S*. *aureus* SSIs reduces 1-year mortality in surgical patients undergoing clean procedures [15]. Eradication treatment not only reduces mortality, it also substantially reduces hospital costs in patients undergoing clean surgery [16]. This shows that a cheap and minimally invasive treatment can greatly reduce patient mortality, as well as substantially lower hospital costs. Other authors have opted for a treat-all strategy instead of the screen-and-treat strategy that was used in this study. While treating all patients and omitting the screening process is a lot cheaper than the screen-and-treat method, the treat-all strategy is associated with a high rate of unnecessary and therefore unethical treatments which increase the probability of the development of bacterial resistance [6, 17].

When applying eradication therapy to *S*. *aureus* nasal carriers who undergo central reconstructive surgery, it might have a greater reduction in SSI rate than patients using eradication therapy before undergoing a peripheral vascular procedure. A possible reason for the difference in effect of treatment between the central reconstructive surgery group and the peripheral surgery group is the higher prevalence of leg ulcers in the peripheral surgery group before and during the surgical procedure. The main reason to perform peripheral vascular surgery is the presence of critical limb ischemia (CLI). In recent literature, the prevalence of patients with CLI and leg ulcers (Fontaine classification stage IV) was estimated as 0.18% [18]. These ulcers may often be polymicrobially infected with gram positive cocci, gram negative rods and anaerobic organisms, which may easily spread to the surgical incision site in the groin area of the affected leg [19]. It is plausible that the introduction of these bacteria to the surgical wound will negate any positive effect the patient could have obtained from eradication therapy for *S*. *aureus*. In the central reconstruction group, the prevalence of leg ulcers, and therefore bacterial influx from the leg, is not higher than in the healthy population, since there is no obstruction of blood flow to the lower limb.

Another explanation for the lack of reduction of SSIs in treated *S*. *aureus*-carriers who undergo peripheral surgery is the recent implementation of a bundle of care in the Amphia Hospital. This bundle of care, which was implemented to reduce SSIs, lowered the incidence of SSIs in patients who underwent vascular surgery by 44% in two years time (from 14.9% to 8.4%) [20]. A further reduction of this already low prevalence of infections might be hard to achieve.

Though it was not the scope of this research, more information regarding *S*. *aureus* eradication therapy and it’s benefits in vascular surgery could be obtained by researching length of hospital stay, graft patency and the incidence of resurgery. Potential bias include selection bias due to the fact that 148 patients were not screened. A number of these patients were not screened due to emergency admissions, which might lead to an over-estimation of the effect of eradication therapy in high-risk patients, since most of our hospital’s acute vascular surgery is performed on patients with a high percentage of comorbidity or patients with a large medical history of vascular illness and/or vascular procedures.

Taking all this into consideration, mupirocin and chlorhexidine-based eradication therapy might provide a minimal invasive, cost-effective means to reduce complications arising from SSIs in vascular surgery.

## Supporting Information

S1 File Database file. (SAV)

S2 File AMOA notice. (PDF)

S3 File Waive of need for ethical approval. (PDF)
