# Supplementary material for: Evaluation of Staphylococcus aureus Eradication Therapy in Vascular Surgery
Source: PLoS One. 2016 Aug 16;11(8):e0161058. doi: 10.1371/journal.pone.0161058 (PMC4986933; doi:10.1371/journal.pone.0161058)
Supplement: S2 File — (PDF) [file pone.0161058.s002.pdf]

LANGENDIJK, Breda
AMOA (Adviescommissie Mensgebonden Onderzoek Amphia)

Bezoekadres Langendijk 75, 4819 EV Breda
Postadres Postbus 90157, 4819 EV Breda

Internet www.amphia.nl

Contactpersoon E. Biemans
Directe telefoon **076- 595 5182**
E-mail **AMOA@amphia.nl**

To whom it may concern

Ons kenmerk EBI/
Datum 29 Februari 2016
Onderwerp **niet WMO-plichtige studie**
Kopie aan

To whom it may concern,

We herewith confirm that it is not required to submit a registry study (Evaluation of Staphylococcus aureus Eradication Therapy in Vascular Surgery), to the AMOA.

Research covered by the Medical Research Involving Human Subjects Act and/or the Embryos Act must be submitted to an accredited Medical Research Ethics Committee (aMREC) for approval before it is carried out.

Submitting of Registry studies is therefore not necessary.

We refer to the site of de CCMO www.ccmo.nl

Yours Faithfully,

Mw. E. Biemans
Ambtelijk secretaris AMOA
