# Supplementary material for: Evaluation of Staphylococcus aureus Eradication Therapy in Vascular Surgery
Source: PLoS One. 2016 Aug 16;11(8):e0161058. doi: 10.1371/journal.pone.0161058 (PMC4986933; doi:10.1371/journal.pone.0161058)
Supplement: S3 File — (PDF) [file pone.0161058.s003.pdf]

MEC-U
MEDICAL RESEARCH ETHICS COMMITTEES UNITED

Medical research Ethics Committees United

Postadres: Postbus 2500, 3430 EM Nieuwegein

Bezoekadressen: Locatie Nieuwegein, Koekoekslaan 1 te Nieuwegein, 030 609 3580
Locatie Eindhoven, Michelangelolaan 2 te Eindhoven, 040 239 8607

E-mail: info@mec-u.nl
Website: www.mec-u.nl

Amphia Ziekenhuis
drs. J.C.M. Langenberg, ANIOS chirurgie
Postbus 90157
4800 RL BREDA

Kenmerk: V.56204/W16.022/hs/cl

Datum: 26-2-2016

Betreft: WMO-plichtigheid onderzoek
W16.022/

Geachte heer Langenberg,

MEC-U heeft de door u ingediende studie met titel: **"Evaluation of Staphylococcus aureus Eradication Therapy in Vascular Surgery"**, waaraan ons registratienummer **W16.022** is toegekend, in goede orde ontvangen.

U heeft de commissie verzocht om te beoordelen of uw studie al dan niet onder de reikwijdte van de Wet medisch-wetenschappelijk onderzoek met mensen (WMO) valt.

De volgende documenten zijn daarbij betrokken:

- aanbiedingsbrief inzake niet WMO d.d. 21-01-2016, ontvangen d.d. 09-02-2016
- onderzoeksprotocol, versie NA, d.d. 21-01-2016

Onderzoek valt onder de reikwijdte van de WMO als voldaan is aan de twee volgende voorwaarden:

1. het is medisch-wetenschappelijk onderzoek, en
2. de proefpersonen worden onderworpen aan handelingen en/of krijgen een gedragswijze opgelegd, zoals bedoeld in de definitie van medisch-wetenschappelijk onderzoek in artikel 1b van de WMO.

Uw studie betreft een retrospectief statusonderzoek waarbij het doel is om te onderzoeken of nasale screening en eradicatie van S. aureus het aantal surgical site infecties door dit micro-orgnaisme vermindert. Hiervoor wordt statusonderzoek verricht onder patiënten die een vaatoperatie hebben ondergaan in de periode van februari 2013 en april 2015 en een controle groep van soortgelijke patiënten in de periode januari – december 2010.
Daar bovengenoemde studie dossieronderzoek betreft, krijgt de proefpersoon geen bepaalde gedragswijze opgelegd en wordt hij niet onderworpen aan handelingen.

Op grond van het bovenstaande ben ik van mening dat deze studie **niet** onder de werking van de WMO valt.
Voor de goede orde deel ik u mee dat uw studie niet op andere punten is beoordeeld.

Vriendelijk verzoek ik u deze studie in te dienen bij de lokale commissie van uw centrum ter verkrijging van een verklaring van geen bezwaar voor uitvoering van de studie in uw ziekenhuis.

Ik verwacht u hiermee voldoende te hebben geïnformeerd.

Met vriendelijke groet,

mw. mr. drs. H. Schoofs, ambtelijk secretaris
Medical research Ethics Committees United (MEC-U)

To whom it may concern,

Referring to your study (reference number W16.022) it is hereby confirmed that the Medical Research Involving Human Subjects Act (WMO) does not apply to the above mentioned study and that therefore an official approval of this study by the MEC-U is not required under the WMO.

Yours sincerely,
on behalf of the Medical Research Ethics Committees United (MEC-U)

Heike Schoofs MA LL.M.
Secretary to the committee
